# Aerosol Jet Printing of Hybrid Ti_3_C_2_T_*x*_/C Nanospheres for Planar Micro-supercapacitors

**DOI:** 10.3389/fchem.2022.933319

**Published:** 2022-07-08

**Authors:** Yu Wu, Aiping Lin, Jidi Zhang, Danjiao Zhao, Lanlan Fan, Cheng Lu, Shufen Wang, Lei Cao, Feng Gu

**Affiliations:** ^1^ Laboratory of Advanced Materials & Manufacturing (LAMM), International Institute for Innovation, Jiangxi University of Science and Technology, Nanchang, China; ^2^ Institute for Process Modelling and Optimization, Jiangsu Industrial Technology Research Institute, Suzhou, China; ^3^ School of Energy and Environment, Southeast University, Nanjing, China

**Keywords:** aerosol jet printing, hybrid structure, nanosphere, MXene, restacking behavior

## Abstract

When utilized in energy devices, the restacking tendency of MXene Ti_3_C_2_T_*x*_ inhibits its electrochemical performance. Using aerosol jet printing (AJP) technology, hybrid Ti_3_C_2_T_*x*_/C nanospheres are synthesized with C nanoparticle-bonded MXene nanosheets, and the restacking of MXene nanosheets is blocked efficiently. The formation mechanism for hybrid Ti_3_C_2_T_*x*_/C nanospheres has been hypothesized, and the Ti_3_C_2_T_*x*_/C is anticipated to assemble and shape along the droplet surface in tandem with the Marangoni flow within the droplet. The planar microsupercapacitor devices generated from these hybrid spherical nanostructures with increased interlayer spacing exhibit exceptional areal capacitance performance. This concept offers a straightforward and effective method for constructing 3D-structured MXene with suppressed self-stacking for diverse high-performance micro energy storage devices.

## 1 Introduction

MXene has been attracting increasing attention because of its good metallicity, relatively large accessible surface area, and the availability of more active sites, endowing it with great potential for applications in energy storage (Ling et al., 2014; Naguib et al., 2014; Cao et al., 2021a; Cao et al., 2021b). However, MXene has a pronounced restacking characteristic with close contact between layers, which greatly reduces the exposed specific surface area and active sites (Xia et al., 2018; Fang et al., 2020; Cao et al., 2021c; Yang et al., 2021). Over the past few years, intensive efforts have been exerted to address this issue. Among them, three-dimensional (3D) structured MXene are expected to expose more active sites with facilitated ion transportation, which is essential for embodying the prominent electrochemical feature of MXene when developing future-related high-performance energy devices (Orangi and Beidaghi, 2020). By applying spherical poly(methyl methacrylate) (PMMA) as a template, a macroporous film of MXene has been developed with a significant increase in specific capacitance performance *(200Fg^−1^ at 10Vs^−1^)* (Lukatskaya et al., 2017). A similar method of sacrificing templates has also been applied to sodium ion storage (Zhao et al., 2017). However, these methods suffer from tedious procedures and time/energy consuming, while residuals are still in a difficult stage to be removed.

Basically, hybridization can be considered as an effective strategy to suppress MXene restacking. For example, knotted carbon nanotubes (CNTs) were developed to support the Ti_3_C_2_ network and restacking could be effectively avoided with enhanced ion accessibility (Gao et al., 2020). Graphene was embedded between Ti_3_C_2_T_*x*_ nanosheets to form a high nanopore connectivity network to facilitate ion transport (Fan et al., 2018). These protocols effectively suppressed the restacking behavior of MXene with enlarged interlayer spacing; however, the resultant hybrid structure was still in a facial form.

Aerosol jet printing (AJP) is a new type of additive manufacturing technology with industrialization prospects. As a non-contact, programmable, and versatile printing technique, the feature size of AJP could reach ∼10 μm (Secor, 2018; Wu et al., 2021), implying the potential for precise preparation of individualized, batched, and miniaturized devices (Mahajan et al., 2013; Jabari and Toyserkani, 2015; Deiner and Reitz, 2017). Currently, AJP has been applied in the fabrication of integrated circuits (Skarzynski et al., 2021), transistors (Cao et al., 2017), memristor (Feng et al., 2019), ring oscillators (Ha et al., 2013), etc. Actually, during the AJP process, the atomized aerosol droplet could be developed as a microreactor mediating the solvent evaporation and solute migration for precisely constructing 3D nanostructures during deposition (Ha et al., 2013). Recently, our group developed a convenient AJP approach for *in situ* synthesis of MXene nanospheres with crumpled and eccentric characteristics (Wu et al., 2022). The shell of the nanosphere was still composed of densely stacked Ti_3_C_2_T_*x*_ nanosheets.

Herein, we developed an effective AJP process for MXene hybrid nanospheres by introducing nanoscale carbon particles (Ti_3_C_2_T_*x*_/C) inhibiting restacking and anchoring neighbouring nanosheets for integrity. The carbon nanoparticles were simply formulated with MXene for the precursor ink. The formation mechanism for the hybrid nanospheres has been proposed tentatively by considering the evaporation-induced migration and assembly process. The derived microsupercapacitor (MSC) device of MXene hybrid nanospheres shows excellent areal capacitance performance of 64.58 mF cm^−2^. This work highlights the great potential of AJP for developing complex nanostructures and broadens the applications of additive manufacturing techniques for miniaturized and intelligent microelectronics.

## 2 Results and Discussion

**FIGURE 1:**
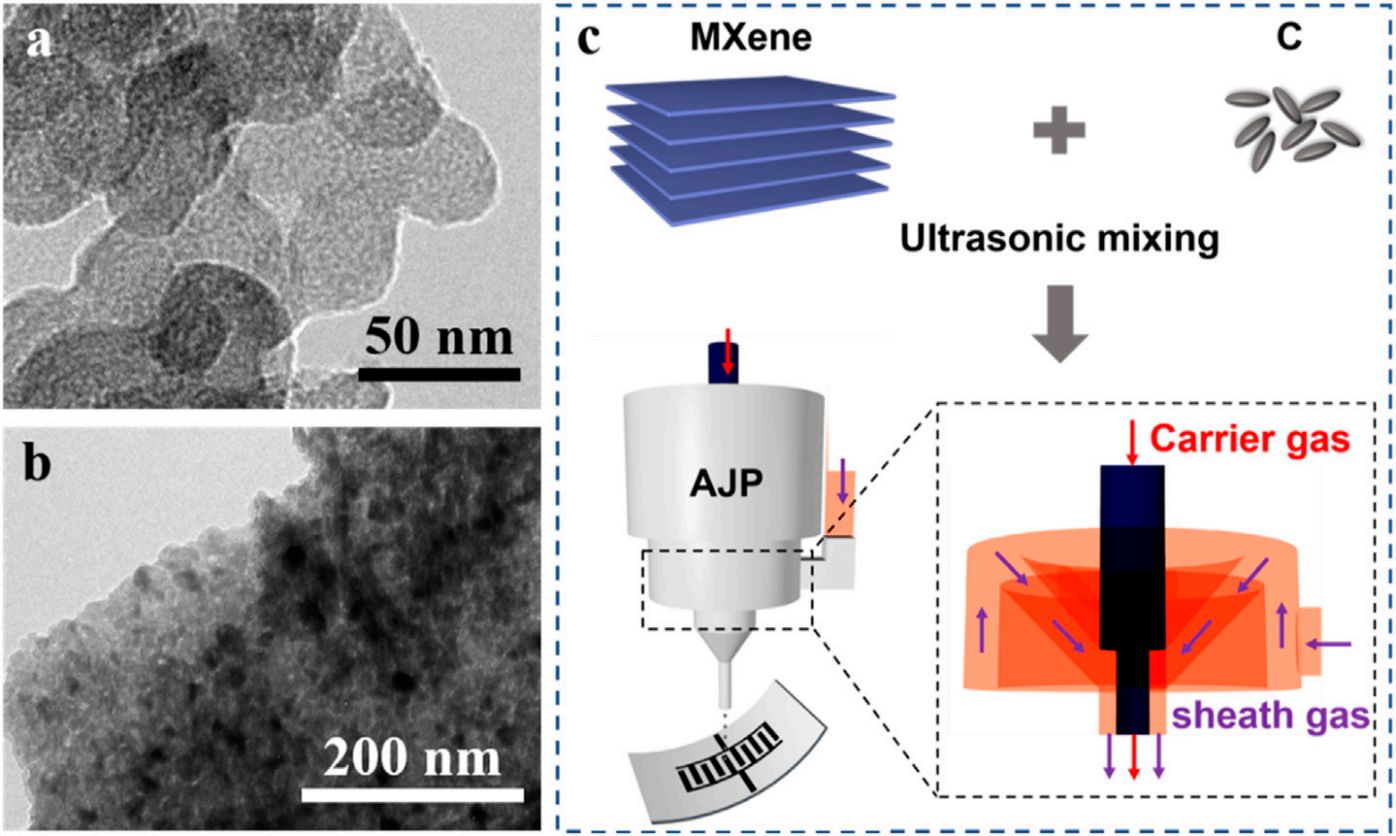
**(A**,**B)** TEM images of the carbon nanoparticles and Ti_3_C_2_T_*x*_/C ink; **(C)** schematic illustration of the AJP process for fabrication of hybrid Ti_3_C_2_T_*x*_/C nanospheres.

The precursor ink was simply formulated by mixing delaminated Ti_3_C_2_T_*x*_ nanosheets and carbon nanoparticles (commercial carbon paint) of different mass ratios in deionized water. The synthesis of the delaminated Ti_3_C_2_T_*x*_ refers previously reported methods, and the details are given in the experimental section (Lukatskaya et al., 2017; Eom et al., 2020; Li et al., 2020). The morphology of the delaminated Ti_3_C_2_T_*x*_ nanosheets is shown in Supplementary Figure S1 (Supporting Information), indicating the MAX phase (Ti_3_AlC_2_) was well etched to a single layer or few-layered nanosheets. Figure 1A shows the transmission electron microscopy (TEM) image of the carbon nanoparticles with a lateral size of 20–30 nm and thickness of 2–3 nm. The X-ray diffraction (XRD) pattern (Supplementary Figure S2, Supporting Information) further verified the carbon of graphite matching information with PDF card 41–1487. Figure 1B show the TEM image of the precursor ink, indicating that the carbon nanoparticles distribute uniformly on the MXene nanosheet surface, which can be further verified by the element mapping results (Supplementary Figure S3, Supporting Information). In our work, the relative mass ratio of Ti_3_C_2_T_*x*_ and C was set at 0:1, 1:0, 1:0.5, 1:1, and 1:2, respectively. Figure 1C schematically illustrates the AJP procedure to fabricate hybrid Ti_3_C_2_T_*x*_/C nanospheres. In case of the aerosol jet printing process, the precursor ink was atomized by an ultrasonic atomizer (1.7 MHz). The generated mist of aerosol droplets (less than 10 μm) was transmitted to the nozzle by a carrier gas of N_2_ and then shaped by a sheath gas of N_2_ jetting out of the nozzle. By confining in the microscale regime of the sheath gas, the mist of aerosol droplets was tremendously focused into a microscale mist jet. The focus ration (FR), which is defined by the sheath gas rate to the carrier flow rate, determines the printing quality. In this work, the FR was fixed at 3 without obvious overspray phenomenon observed. The focused aerosol jet was subjected on the oxygen plasma–treated polyethylene terephthalate (PET) surface. The deposition temperature was 100°C for accelerating the solute migration.

**FIGURE 2:**
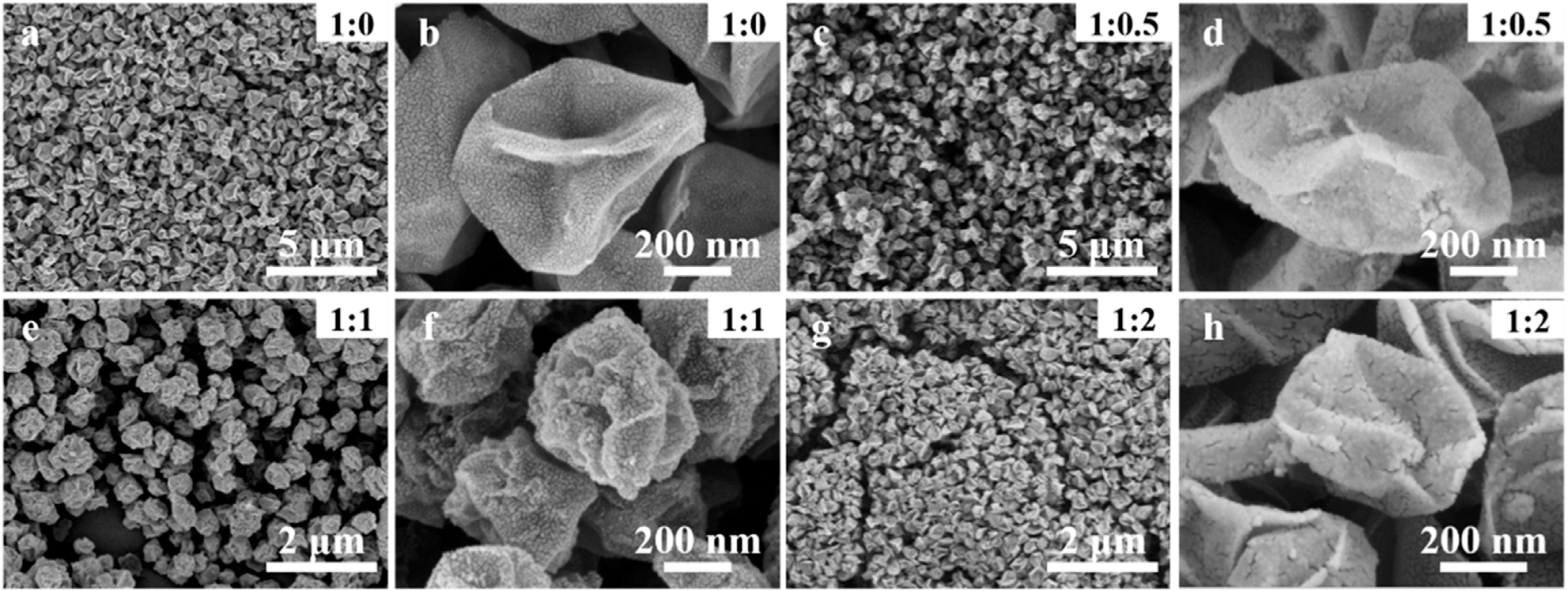
SEM images of the printed hybrid Ti_3_C_2_T_*x*_/C nanospheres with different mass ratios of MXene and carbon nanoparticles. **(A,B)** Pristine MXene nanospheres; **(C,D)** hybrid MXene nanospheres with mass ratio of 1:0.5; **(E,F)** hybrid MXene nanospheres with mass ratio of 1:1; **(G,H)** hybrid MXene nanospheres with mass ratio of 1:2.

**FIGURE 3:**
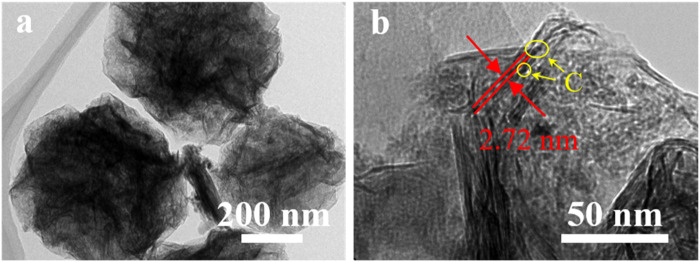
HRTEM images of the printed hybrid Ti_3_C_2_T_*x*_/C nanospheres showing the enlarged interlayer distance. The yellow circles mark the embedded carbon nanoparticles.

The morphology and microstructure of the printed patterns were characterized by SEM and TEM, indicating the formation of crumpled nanospheres with ridges or wrinkles while the pristine facial MXene nanosheets were not detected (Figures 2A,B). The size of the formed spheres is ∼500 nm, independent of carbon nanoparticles added. With the introduction of carbon nanoparticles, the spheres surface become rough and the carbon nanoparticles could be clearly observed (Figures 2C–F). When excessive carbon nanoparticles added (mass ratio of 1:2), a rather dense film composed of closely bonded nanospheres were formed (Figure 2G). It is found that the carbon nanoparticles are distributed homogeneously in the resultant hybrid products in case of the formulation of the precursor ink by simply mixing these two components. Due to the hydrophilic nature, the Ti_3_C_2_T_*x*_ nanosheets could be dispersed in the solvent of water homogeneously. The negatively charged surface of MXene is assumed as the main reason for absorbing the carbon nanoparticles for formation of the hybrid structure. Under the ultrasonic condition for atomization, the dispersed carbon nanoparticles are prone to be adsorbed on the MXene nanosheets surface. During the assembly of Ti_3_C_2_T_*x*_/C for the resultant spherical nanostructure, the adsorbed carbon nanoparticles could effectively inhibit the closely restacking tendency of MXene nanosheets. From Figure 3B, the embedded carbon nanoparticles could be clearly observed and the interlayer distance is enlarged greatly up to 2–3 nm, consistent with the size of the carbon nanoparticles. The interlayer distance is obviously larger than its pristine MXene counterpart (less than 1 nm) (Supplementary Figure S4, Supporting Information). Here, the adsorbed carbon nanoparticles could also function as binders to bridge neighbouring nanosheets for integrity, which is particularly important for optimizing the electrochemical performance.

**FIGURE 4:**
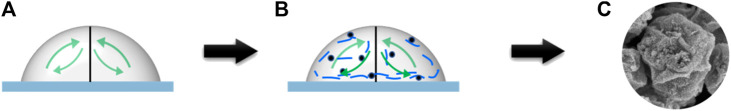
Schematic illustration of the formation mechanism of hybrid MXene/C nanospheres. **(A)** Marangoni flow occurs in the deposited droplet due to the temperature gradient on heat substrate. **(B)** Carbon nanoparticles-anchored MXene nanosheets migrating from the interior to the edge; **(C)** MXene/C precipitating at the edge and shaping along the droplet surface for a spherical structure.

For a microscale droplet containing Ti_3_C_2_T_*x*_/C components depositing on the heat PET surface, which is supposed to offer an isotropical evaporation regime, the temperature gradient along the liquid-vapor interface between the apex and the bottom of the drop causes a Marangoni flow inside the droplet (Girard et al., 2008). The evaporation of water from the edge is replenished by water from the interior, carrying Ti_3_C_2_T_*x*_/C toward the edge by the Marangoni flows. As the evaporation progresses, the liquid/solid/gas three phase contact line (TCL) gradually recedes. The transmitting Ti_3_C_2_T_*x*_/C are easily precipitated on the substrate surface at the edge and further shaped along the droplet surface during solvent evaporation to form a spherical structure with eccentric features by referring velocity field analysis of sessile water droplets on heat substrate, although Ti_3_C_2_T_*x*_ MXene is generally resistant to bending due to high bending rigidity (Wu et al., 2021). The bending of Ti_3_C_2_T_*x*_ nanosheets are expected to be triggered by the sonication for the aerosol droplets generation with stress inequality, which has been evidenced during the synthesis of graphene nanoscrolls previously reported (Savoskin et al., 2007). Moreover, Laplace pressure, which is correlated to the curvature radius (R) of the droplet, (Wu et al., 2022), increased greatly along with the decreasing droplet size on the heat substrate due to solvent evaporation for further bending the MXene nanosheets. With the formation of eccentric nanospheres, the anchored carbon nanoparticles are liable to bond neighbouring nanosheets with favorable interlayer distances. The formation mechanism of the hybrid Ti_3_C_2_T_*x*_/C nanospheres is schematically illustrated in Figure 4.

**FIGURE 5:**
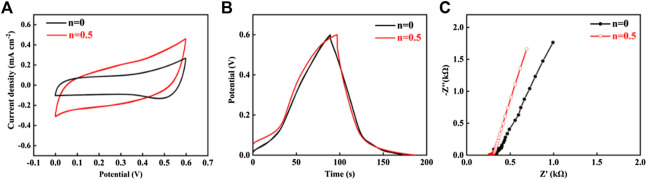
**(A)** CV curves of the MSC devices of hybrid MXene/C nanospheres with different mass ratios at a scan rate of 20 mV s^−1^. **(B)** GCD profiles at a current density of 0.2 mA cm^−2^. **(C)** EIS of the microdevice of hybrid MXene/C and its pristine MXene counterpart.

The electrochemical performance of the hybrid MXene/C nanospheres was tentatively investigated by printing interdigital microelectrodes via the AJP process. The interdigital microelectrode was realized by multiple printing passes of feature size of 200 μm. In view of the influence of electrode configuration (e.g., line length, width, thickness and gap distance) on the electrochemical performance, in this work, the interdigital microelectrodes of hybrid MXene/C nanospheres were printed with line width of 200 μm, thickness of 95 μm, and gap distance of 170 μm, respectively, after 50-time printing passes and the working area was estimated to be 3.7 mm × 3.8 mm (Supplementary Figure S5, Supporting Information). The MSC devices were fabricated by applying a gel electrolyte based on poly (vinyl alcohol) (PVA)/H_2_SO_4_ onto the interdigital microelectrodes with labeling as MSC-n, where n designates the relative mass ratio of carbon nanoparticles (Supplementary Figure S6, Supporting Information). Figure 5A shows the cyclic voltammogram (CV) curves of the MSC devices at n values of 0 and 0.5. The quasi-rectangular CV curves indicate that the presence of pseudocapacitance and electric double layer capacitance behavior (Cao et al., 2018; Cao et al., 2019; Das et al., 2020). Figure 5B shows the GCD curves of the MSC devices at a current density of 0.2 mA cm^−2^ and the approximately symmetrical curves indicates good reversibility and the non-linear curves in the potential during both charge and discharge half-cycles shows a typical feature of a hybrid supercapacitor (Yu et al., 2020). The areal capacitance was estimated to be 33.14 mF cm^−2^, for MSC-0.5 device, obviously larger than the pristine MXene device. The addition of carbon nanoparticles is clear to greatly enhance the electrochemical performance of MXene-based devices. Noted that although the quantity of carbon nanoparticles is comparable to the MXene when formulating the precursor inks, actually the atomized aerosol droplets contain minimal carbon nanoparticles due to the fact that only the supernatant of the ink containing MXene nanosheet of suitable lateral size (less than the aerosol droplet size) could be successfully atomized and large quantity of carbon nanoparticles were captured by large MXene nanosheets or agglomerated under the sonication. The TEM and SEM images shown in Figures 1–3 could verify this conjecture with dotted carbon nanoparticles present. Therefore, the contribution of carbon nanoparticles on the areal capacitance of the hybrid system could be neglected. The improved areal capacitance mainly arises from the spherical nanostructures with broadened interlayer distances, which is expected to favor the ionic transportation for a promising electrochemical performance. By optimizing the mass ratio of carbon nanoparticles (n = 1), the assembled device exhibited an optimal areal capacitance of 46.95 mF cm^−2^, exceeding the values of microscale devices fabricated by other additive manufacturing techniques, e.g., direct writing and inkjet printing reported previously (Quain et al., 2019; Zhang et al., 2019). The electrochemical impedance spectroscopy (EIS) measurements were applied to explore the electronic/ionic transport behaviors of the microelectrodes. As shown in Figure 5C, the Nyquist plot at the high-frequency region of the hybrid microelectrode indicates a faster ion diffusion in comparison to pristine MXene electrode. The results demonstrate that the hybrid microelectrode could accommodate more electrochemically active sites and enable the electrolyte to permeate more readily, enhancing the capacitive performance.

## 3 Conclusion

In conclusion, hybrid Ti_3_C_2_T_*x*_/C nanospheres with crumpled and eccentric features have been successfully developed by a convenient AJP approach. The addition of carbon nanoparticles could effectively inhibit the closely restacking of MXene nanosheets during the assembly process. Meanwhile, the anchored carbon nanoparticles could act as binder to bridge neighbouring nanosheets and nanospheres for integrity. Arising from the temperate gradient-derived Marangoni flow, the Ti_3_C_2_T_*x*_/C could be easily transported and further shaped along the droplet surface with the solvent evaporation. Due to the special hybrid spherical characteristic, the fabricated MSC devices derived from the hybrid Ti_3_C_2_T_*x*_/C nanospheres demonstrate faster ion diffusion and excellent areal capacitance. Accordingly, the areal capacitance is greatly enhanced in comparison to Ti_3_C_2_T_*x*_ counterpart. The AJP approach developed in this work highlight its potential for developing future high-performance microdevices with capabilities of structure modulation at multiscale.

## 4 Materials and Methods

*Preparation of delaminated Ti*_*3*_ *C*_*2*_ *T*_*x*_ *nanosheets:* Typically, LiF (0.5 g, Aladdin) was dissolved in HCl (9 M, 10 ml, Aladdin) and stirred until completely clear at room temperature. Ti_3_AlC_2_ powder (0.5 g, 11 Technology) was slowly added to the aforementioned solution accompanied by vigorous stirring in an ice bath. After being stirred at 40 °C for 48 h, the mixture was washed with deionized water and centrifuged at 7500 rpm for 5 min until the pH of the supernatant was ∼6. Subsequently, the obtained sediment was dispersed in deionized water, shaken vigorously, and then sonicated for 1 h. The resulting mixture was centrifuged at 3500 rpm for 1 h, and the supernatant was transferred for freeze-drying to obtain the delaminated Ti_3_C_2_T_*x*_ nanosheets.

*Preparation of Ti*_*3*_ *C*_*2*_ *T*_*x*_ */C ink:* The precursor ink was formulated by mixing delaminated Ti_3_C_2_T_*x*_ nanosheets and carbon nanoparticles (Bare Conductive Ltd.) of different mass ratios in deionized water. After shaking vigorously, the Ti_3_C_2_T_*x*_/C ink is ready for printing.

*Printing of Ti*_*3*_ *C*_*2*_ *T*_*x*_ */C interdigital microelectrodes:* A commercial aerosol jet printer (WE-HMP, WE Electronics) was applied for the printing process. The interdigital patterns were designed by CAD software, which can be readable by the printer. The Ti_3_C_2_T_*x*_/C ink was atomized into droplets with the aid of an ultrasonic atomizer (1.7 MHz). The nozzle diameter was 500 μm and the stand-off height was ∼8 mm. When the carrier gas (N_2_) and the sheath gas (N_2_) were set to 50 and 150 sccm, respectively, the aerosol beam was focused without obvious overspray. The substrate, polyethylene terephthalate (PET), was cleaned with ethanol, dried by, and then plasma-treated for 400 s (VP-R, SunJune) before use. The printing speed was fixed at 10 mm s^−1^. The deposition temperature was set to 100 °C. All the interdigital microelectrodes were obtained after 50-time printing passes.

*Fabrication of Ti*_*3*_ *C*_*2*_ *T*_*x*_ */C MSCs:* The poly(vinyl alcohol) (PVA)/H_2_SO_4_ electrolyte was prepared by dissolving 3 g of PVA (87–89% alcohol solubility) in 15 mL deionized water. After being stirred at 60 °C for 15 min, another 15 mL of deionized water was added, accompanied by being stirred at 85 °C for 3 h until the solution was completely clear and transparent. After cooling to room temperature, 3 mL of sulfuric acid (98%, Aladdin) was added dropwise for 1 h. In addition, two silver wires were connected separately with two electrodes by conductive silver enamel. After the enamel dried absolutely, the electrolyte gel was coated onto the interdigital electrodes.

*Materials Characterizations:* The morphologies and microstructures were characterized by a transmission microscope (TEM, Titan G260-300) and a scanning electron microscopy (SEM, Zeiss Gemini 300) together with an energy-dispersive X-ray spectroscope (EDX, Zeiss Smart). X-ray diffraction patterns (XRD) were obtained by using a PIGAKV Ultima IV X-ray diffractometer with a Cu K_α_ radiation source (*λ* = 0.15418 nm).

*Electrochemical Measurement:* Cyclic voltammetry (CV), galvanostatic charging/discharging (GCD), and spectroscopy (EIS) were conducted on an electrochemical workstation (Princeton, Versa STAT 4). The areal capacitance of the MSCs was calculated based on the GCD results as following: C_A_ = It/(SΔV), where C_A_ (mF cm^−2^) refers to the areal capacitance, I (A) refers to the discharge current, t (s) refers to the discharge time, S (cm^2^) refers to the geometric area of the electrode, and ΔV (V) refers to the working potential window.

## Data Availability

The original contributions presented in the study are included in the article/Supplementary Material; further inquiries can be directed to the corresponding author.
