# Supplementary material for: Aerosol Jet Printing of Hybrid Ti3C2T x /C Nanospheres for Planar Micro-supercapacitors
Source: Front Chem. 2022 Jul 8;10:933319. doi: 10.3389/fchem.2022.933319 (PMC9304703; doi:10.3389/fchem.2022.933319)
Supplement: Supplementary file 1 [file DataSheet1.docx]

**Supplementary Information**

Aerosol Jet Printing of Hybrid Ti_3_C_2_T*_x_*/C Nanospheres for Planar Micro-supercapacitors

**Yu Wu^1, a^, Aiping Lin^1, a^, Jidi Zhang^2^, Danjiao Zhao^1^, Lanlan Fan^1^, Cheng Lu^2^, Shufen Wang^1,^*, Lei Cao^1^, and Feng Gu^1, 2, 3^***

^1^ Laboratory of Advanced Materials & Manufacturing (LAMM), International Insititute for Innovation, Jiangxi University of Science and Technology, Nanchang, Jiangxi 330013, China

^2^ Institute for Process Modelling and Optimization, Jiangsu Industrial Technology Research Institute, Suzhou, Jiangsu 215123, China

^3^ School of Energy and Environment, Southeast University, Nanjing, Jiangsu 210096, China

^a^ These authors contributed equally.

*** Correspondence:**

Corresponding author.

E-mail: feng.gu@jxust.edu.cn


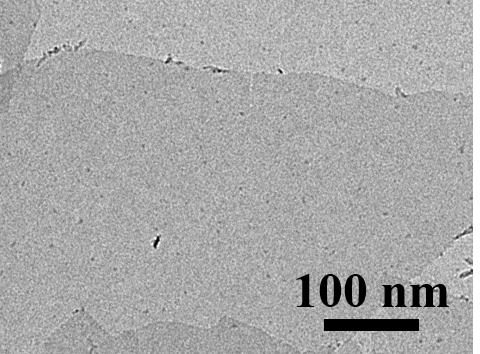


Figure S1. TEM image of the delaminated Ti_3_C_2_T*_x_* nanosheets.


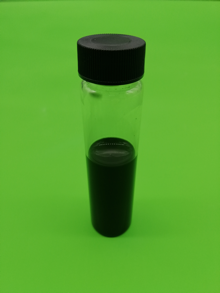




Figure S2. XRD pattern of the commercially available carbon nanoparticles (Inset shows the dispersion of carbon nanoparticles in water).


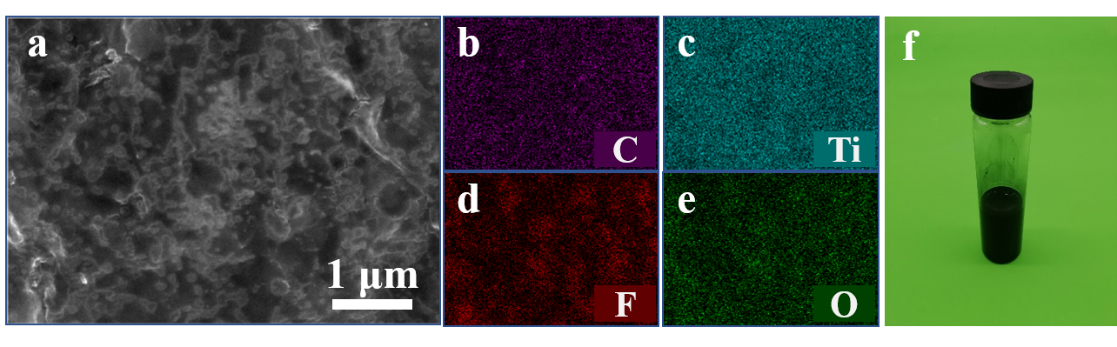


Figure S3. **a)** SEM image of the precursor ink containing MXene and carbon nanoparticles showing the carbon nanoparticles anchoring on the nanosheet surface. **b-e)** Elemental mapping showing the homogeneous distribution of C and Ti elements. **f)** Precursor ink containing MXene and carbon nanoparticles.


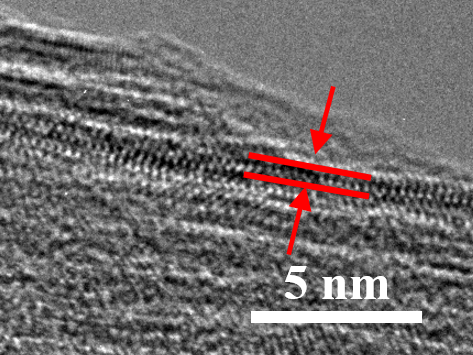


Figure S4. TEM image of pristine MXene nanospheres.


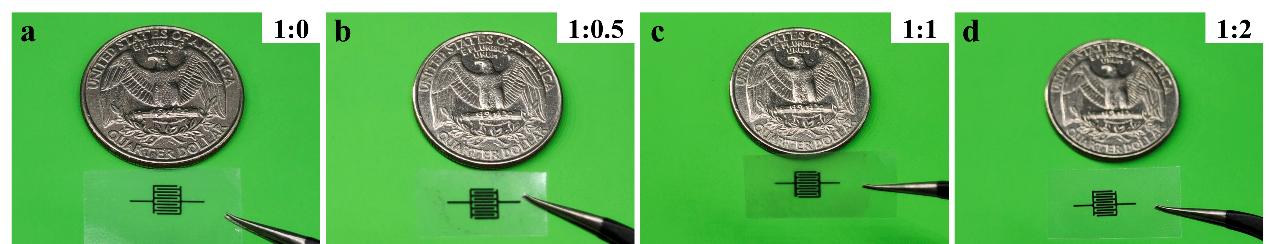


Figure S5. Photos of the AJP-derived interdigital microelectrode of hybrid Ti_3_C_2_T_x_/C nanospheres with different mass ratios of Ti_3_C_2_T*_x_* and carbon nanoparticles: **a)** 1:0, **b)** 1:0.5, **c)** 1:1 and **d)** 1:2.


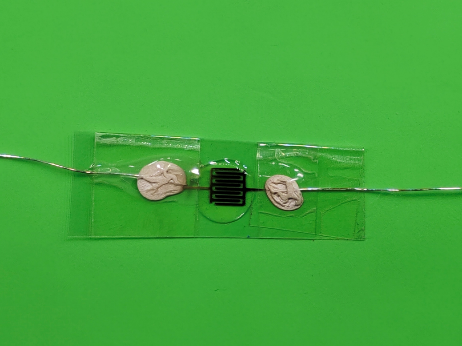


Figure S6. Photos of the fabricated MSC device.
